# Fetal dependency on maternal fatty acids: a pilot study in human pregnancies using the natural abundance variation of ^13^C

**DOI:** 10.1017/S0007114524003222

**Published:** 2025-01-28

**Authors:** Simonato Manuela, Verlato Giovanna, Visentin Silvia, Erich Cosmi, Anna Sartori, Pieter Sauer, Alessio Correani, Cogo Paola, Carnielli Virgilio

**Affiliations:** 1 Department of Women’s and Children’s Health, University of Padova, Padova, Italy; 2 Pediatric Research Institute ‘Citta’ della Speranza’, Critical Care Biology and PCare Laboratories, Padova, Italy; 3 Department of Pediatrics, University of Groningen, Beatrix Children’s Hospital/UMCG, Groningen, Netherlands; 4 Department of Odontostomatologic and Specialized Clinical Sciences, Polytechnic University of Marche, Ancona, Italy; 5 Department of Medicine, Division of Pediatrics, S. Maria della Misericordia University Hospital, University of Udine, Udine, Italy; 6 Division of Neonatology, Mother and Child Department, G. Salesi University Hospital, Ancona, Italy

**Keywords:** Carbon-13, Natural abundance, Placenta, Fetal biosynthesis, Fatty acids

## Abstract

The extent of *de*-*novo* biosynthesis of non-essential fatty acids (FA) and the endogenous biosynthesis of long chain PUFA in human fetuses remain largely unknown. We used natural variations in the ^13^C:^12^C (δ ^13^C) of plasma phospholipids of the woman at delivery and of cord blood to infer fetal biosynthesis of FA. We studied thirty-nine mother–fetus pairs with uncomplicated pregnancies and term delivery. Eighteen women were supplemented with DHA, from pregnancy week 20 until delivery, sourced from an algae (*n* 13) or fish oil (*n* 5), each with slightly different ^13^C content. Twenty-one women did not receive DHA supplementation. We measured the δ ^13^C value of selected phospholipid FA (C16:0, C18:0, C18:1*n*-9, C18:2*n*-6, C20:4*n*-6 and C22:6*n*-3) in maternal and cord plasma samples at delivery using isotope ratio MS. We found significant linear correlations for δ ^13^C values of FA between mothers and their fetuses (C16:0, *r* = 0·8535; C18:0, *r* = 0·9099; C18:1*n*-9, *r* = 0·8079; C18:2*n*-6, *r* = 0·9466; C20:4*n*-6, *r* = 0·9257 and C22:6*n*-3, *r* = 0·9706). Women supplemented with algal DHA had significantly lower DHA δ ^13^C values in their plasma phospholipids than those supplemented with fish DHA or those who did not receive DHA supplementation (*P* < 0·001). There was no significant difference in δ ^13^C values of FA between women at delivery and their fetuses. These findings strongly suggest that the human fetus is highly dependent on the placental transport of maternal plasma FA, particularly DHA. The limited fetal biosynthesis of major FA emphasises the crucial role of maternal nutrition and placental well-being in fetal development.

Fatty acids (FA) are required by the developing fetus to support rapid cellular growth and function. Deposition of lipids increases exponentially during gestational age, reaching around 7 g/d just before birth^(1)^. Among these, DHA (C22:6*n*-3) and arachidonic acid (20:4*n*-6; AA) are crucial for optimal fetal growth and the development of important organs such as the retina and brain^(2,3)^.

From the beginning of pregnancy, the woman adjusts her metabolism to support the nutritional needs of the fetal–placental unit by increasing levels of nearly all plasma lipid classes^(4)^. As the fetus develops, the placenta actively transports substantial amounts of FA from the mother to the fetus^(5)^, particularly during the third trimester when brain tissue rapidly increases in cell size and number^(6)^. Higher amounts of DHA and AA acids and lower amounts of their precursors, alpha linolenic acid (C18:3*n*-3) and linoleic acid (C18:2*n*-6, LA), are found in fetal plasma compared with maternal plasma^(7)^. It has been estimated that the rate of fetal accretion for AA and DHA between 35 and 40 weeks of gestational age is 92 and 42 mg/d, respectively^(8)^.

While the mechanism of FA transfer across the placenta is well established^(9,10)^, our understanding of the contribution of maternal FA supply to the fetus and the extent of the fetal *de novo* FA biosynthesis (lipogenesis) remain rather limited.

Some information on the placental transfer of FA comes from an *in vivo* short study using palmitic acid (C16:0, PA), oleic acid (C 18:0, OA), LA and DHA, uniformly labeled with ^13^C in both control and gestational diabetes mellitus pregnant women. These FA were administered orally 12 h before elective cesarean section. The ^13^C enrichment was then measured in maternal plasma, cord blood and the placenta. The authors found that 0·5 % of administered FA were detectable in cord blood, except for DHA which was enriched by 3·5 %^(11)^. The preferential placental transfer of DHA over AA, LA and alpha linolenic possibly reflects the high demand for DHA in the growing fetus^(12)^. Moreover, although the fetal ability to desaturate and elongate LA to AA was demonstrated in 9-term infants^(13)^, endogenous biosynthesis appears less efficient in the case of DHA chain elongation from alpha linolenic^(14)^.

Information on the biosynthesis of saturated and unsaturated non-essential FA by the human fetus is limited to older *in vitro* studies of human subcutaneous fetal tissue and rat fetuses^(15)^. It is known that FA synthesis in adipose tissue begins to increase continuously from as early as 10 weeks of gestation,^(16)^ and that rat fetuses synthesise FA *de novo* using maternal glucose as a primary precursor^(17)^. Given the undisputed importance of the diet of the pregnant woman and of DHA supplementation during pregnancy^(18,19)^, further understanding of fetal lipid synthesis with respect to maternal DHA and FA placental transfer remains of importance.

Our clinical research group has recently utilised the natural variations in the ^13^C/^12^C (δ ^13^C) value to measure the contribution of DHA from an algal source to the plasma DHA pool in pregnant women^(20)^. With this approach, under the premise that the ^13^C content of the nutrient of interest must be different from the isotopic background, it was possible to trace the metabolism of a given metabolite and separate the dietary from the endogenous component in biological samples^(21–23)^.

In this study, we applied the ^13^C natural abundance approach to compare the δ ^13^C values of selected maternal and fetal FA, to infer the placenta transfer from the mother to the fetus. We aimed to determine whether *de*-*novo* biosynthesis of non-essential FA and the biosynthesis of long-chain PUFA *via* chain elongation and desaturation of essential FA occur in the human fetus and whether these potential processes contribute to the overall fetal FA turnover.

## Methods

The study group consisted of thirty-nine mother–fetus pairs. Ten were part of an old study conducted in Holland,^(21)^ and twenty-nine were studied in Italy. Twenty-nine out of the thirty-nine mother–fetus pairs were part of previous studies by our group^(20,21)^.

Eighteen Italian pregnant women were supplemented with 200 mg/d of DHA from pregnancy week 20 until delivery (DHA^+^ group), while 21 women did not take any DHA supplementation (DHA^–^ group).

In the supplement, DHA was in the form of triglyceride oil from algal (*n* 13) or fish (*n* 5) origin. Participation in the study was voluntary, and informed consent was obtained from all subjects. This study was conducted according to the guidelines laid down in the Declaration of Helsinki and all procedures involving human subjects/patients were approved by the local ethical committee of Padova Hospital (protocol number 1333P) and of Sophia Children’s Hospital, Erasmus University, Rotterdam.

The inclusion criteria were single pregnancy, aged 18–40 years, uncomplicated pregnancies, and term deliveries.

### Blood samples

Cord blood samples were obtained at delivery, whereas maternal venous blood samples were obtained from the antecubital vein within 2 h from delivery. Blood collected in EDTA tubes was centrifuged within 2 h of collection, and the plasma aliquots were stored for a maximum of 10 years, at −80°C in tubes containing pyrogallol as an antioxidant.

### Sample preparation

Lipids were extracted from 100 µl of plasma samples using a chloroform–methanol mixture with butylated hydroxytoluene as an antioxidant by the Folch method^(24)^. Lipid class separation and phospholipid isolation were reported previously^(25)^.

Briefly, the lipid extract was resolved in classes by thin-layer chromatography, and the phospholipid fraction was hydrolysed with HCl–methanol. The resulting methyl ester FA were extracted with hexane. The hexane layer was collected in vials for gas chromatographic analysis.

### Gas chromatography analysis

Gas chromatography analysis was performed as previously described^(20)^ and FA composition was reported as the percentage of each FA relative to the total phospholipid FA (mol %).

### δ^13^C of plasma phospholipids fatty acids

The δ^13^C value of FA methyl esters from plasma phospholipids was analysed by using a gas chromatography-combustion interface isotope ratio mass spectrometer (GC-C-IRMS, Delta V Thermo Fisher Scientific, Bremen, Germany) as previously reported^(20)^. The system was externally calibrated with certified standard mixtures F8–2 for FA methyl ester (even chain FA methyl and ethyl esters from n-C14:0 to n-C20:0), obtained from Arndt Schimmelmann.

Carbon isotopic analysis was performed in triplicates. The values of δ^13^C were expressed in milliUrey (mUr)^(26)^. Each mUr is representative of a one per mill (1 in 1000, ‰) change in the δ^13^C with respect to the Vienna Pee Dee Belemnite international reference standard:

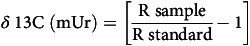




where ‘R’ is the ratio of the heavy to light isotope in the sample or standard.

### Statistical analysis

Data were presented as mean (standard deviation) (sd) or as median and interquartile range (25°th–75°th percentile). Pearson correlation was used to assess the association between maternal and fetal δ^13^C of FA.

Inter-group comparisons were performed using the Mann–Whitney test, while intra-group comparisons were conducted with the Wilcoxon test.

All tests were two-sided, and a *P*-value < 0·05 was considered statistically significant. Statistical analysis was performed using PASW Statistics 18.0 (IBM Corp).

## Results

The twenty-eight Italian women were recruited in Padua, Italy between 2015 and 2017, and the 11 Dutch women were recruited in Rotterdam, The Netherlands.

Participants’ characteristics were: body weight of 63 (sd 9) kg, weight gain during pregnancy of 11 (sd 3) kg, length of gestation 40 (sd 1) weeks, and newborn birth weight of 3453 (sd 368) g.

The DHA^+^ group was composed of 18 Italian women, whereas there were 10 Italian and 11 Dutch women in the DHA^–^ group.

### Fatty acid composition and δ^13^C value of DHA supplements

Detailed composition of DHA supplement (from algae or fish) has been determined and recently published^(20)^ and is reported in Table 1. The percentage of DHA of the two preparations was not different being 45·7 and 45·8 mol% in fish and algae supplements, respectively (*P* = 0·80), whereas it was significantly different in the δ^13^C DHA value (–25·3 (sd 0·2) *v*. −15·8 (sd 0·2), *P* < 0·001).

**Table 1.** Fatty acid composition and δ^13^C of DHA supplements

	Fish group (mol%)		Algae group (mol%)			Fish group δ^13^C (mUr)		Algae group δ^13^C (mUr)		
FA	Mean	s d	Mean	sd	*P*	Mean	sd	Mean	sd	*P*
8:0	0·94	0·10	0·83	0·12	0·37	–	–	–	–	–
10:0	0·56	0·09	0·45	0·11	0·27	–	–	–	–	–
12:0	–	–	–	–	–	–	–	–	–	–
14:0	3·64	0·04	0·98	0·08	< 0·001	–25·0	0·2	–	–	–
16:0	15·56	0·15	22·34	0·05	0·007	–24·0	0·2	–9·3	0·2	< 0·001
18:0	4·50	0·09	2·13	0·10	< 0·001	–23·7	0·2	–	–	–
20:0	0·59	0·13	0·29	0·02	0·007	–	–	–	–	–
16:1*n*-7	3·44	0·05	0·43	0·04	< 0·001	–24·0	0·2	–	–	–
18:1*n*-9	9·76	0·09	21·64	0·21	< 0·001	–24·0	0·3	–27·3	0·1	< 0·001
18:2*n*-6	1·46	0·08	2·70	0·09	< 0·001	–	–	–27·9	0·1	–
18:3*n*-6	0·65	0·09	–	–	–	–	–	–	–	–
18:3*n*-3	–	–	–	–	–	–	–	–	–	–
20:4*n*-6	3·01	0·13	0·65	0·03	< 0·001	–26·3	0·2	–	–	–
20:5*n*-3	7·53	0·21	–	–	–	–25·1	0·3	–	–	–
22:5*n*-3	1·97	0·01	–	–	–	–	–	–	–	–
22:6*n*-3	45·75	0·07	45·82	0·32	0·80	–25·3	0·2	–15·8	0·2	< 0·001

Values are means (sd), N = six different batches.

Fatty acid composition is expressed as the percentage of fatty acid in total fatty acids. δ^13^C, carbo*n*-13 isotopic abundance.

*P* determined by independent *t* test.

Table from Simonato M, Visentin S, Verlato G, *et al.* DHA turnover in pregnant women using the natural abundance variation of ^13^C: a pilot study. British Journal of Nutrition. 2023; 129(2):240–246. doi:10.1017/S000711452200108.

### Fatty acids composition of plasma phospholipids

The quantitative data of plasma phospholipids are available for twenty-eight out of thirty-nine mother–fetal dyads. Unfortunately, due to a computer system crash, we lost the chromatogram of the phospholipids FA belonging to the Dutch group. It was not possible to provide information on these samples, as there was not enough plasma left to repeat the analysis.

The phospholipid FA composition data are reported in Table 2. There was no significant difference in total phospholipid content between the DHA^+^ and DHA^–^ groups in the mother and fetus.

**Table 2.** Plasma phospholipid fatty acid composition (mol %) in DHA^+^ and DHA^–^ pregnant women and their fetuses (Median values and interquartile ranges)

	W. DHA^+^		W. DHA^–^			F. DHA^+^		F. DHA^–^					(F.-M.) DHA^+^		(F.-M.) DHA^–^		
FA (mol %)	Median	25th–75th percentile	Median	25th–75th percentile	*P* ^*^	Median	25th–75th percentile	Median	25th–75th percentile	*P* ^*^	*P* ^†^ DHA+	*P* ^†^ DHA–	Median	25th–75th percentile	Median	25th–75th percentile	*P* ^*^
12:0	0·09	0·04–0·12	0·09	0·05–0·13	0·832	0·27	0·10–0·44	0·25	0·15–0·33	0·555	0·001	0·007	016	0·01–0·34	0·15	0·09–0·19	0·654
14:0	0·68	0·49–0·75	0·62	0·55–0·74	0·689	0·69	0·60–0·90	0·67	0·49–0·79	0·494	0·145	0·386	0·13	−0·16–0·31	0·08	−0·12–0·18	0·621
16:0	37·56	35·41–38·30	38·27	36·10–39·47	0·654	37·05	35·00–38·78	36·99	34·96–37·79	0·689	0·420	0·508	−0·97	−3·13–2·28	−2·04	−2·80–2·35	0·869
16:1*n*-7	0·98	0·74–1·29	0·86	0·52–1·27	0·555	1·06	0·78–1·65	0·96	0·75–1·19	0·308	0·122	0·646	0·23	−0·23–0·76	0·16	−0·34–0·37	0·524
18:0	8·51	7·70–9·56	8·94	7·59–10·47	0·494	12·15	10·91–15·40	14·26	11·64–15·98	0·577	0·000	0·013	4·49	1·99–7·30	5·51	3·45–6·15	0·981
18:1-*n*9	9·90	8·24–10·50	9·49	7·55–11·48	0·944	6·53	5·79–7·34	6·34	5·89–7·50	1·000	0·000	0·003	−2·84	−4·31 – −2·03	−2·98	−4·46 – −1·10	0·981
18:1-*n*7	1·38	1·24–1·65	1·36	1·14–1·48	0·322	2·33	1·97–2·62	2·34	2·06–2·62	0·654	0·002	0·005	0·78	0·59–1·31	1·07	0·91–1·35	0·121
18:2-*n*6	19·19	16·64–20·66	20·99	19·02–22·62	0·057	6·36	5·87–7·83	7·76	6·50–9·22	0·045	0·000	0·005	−12·43	−13·46 – −10·65	−13·23	−15·13 – −11·05	0·226
18:3-*n*6	0·11	0·06–0·13	0·10	0·08–0·13	1·000	0·00	0·00–0·15	0·11	0·09–0·14	0·226	0·278	0·575	−0·04	−0·10–0·05	0·02	−0·03–0·04	0·245
18:3-*n*3	0·14	0·12–0·18	0·13	0·09–0·16	0·621	0·00	0·00–0·00	0·00	0·00–0·00	0·524	0·000	0·005	−0·14	−0·18 – −0·11	−0·14	−0·17 – −0·08	0·654
20:0	0·37	0·34–0·51	0·38	0·31–0·43	0·832	0·59	0·53–0·70	0·75	0·54–0·94	0·160	0·000	0·005	0·23	0·06–0·33	0·42	0·16–0·49	0·089
20:2-*n*6	0·28	0·23–0·35	0·31	0·23–0·39	0·524	0·25	0·20–0·40	0·32	0·24–0·36	0·464	0·678	0·646	0·01	−0·11–0·18	−0·02	−0·07–0·05	0·832
20:3-*n*6	3·12	2·74–3·58	2·90	2·42–3·74	0·689	4·02	3·49–5·21	4·19	4·01–4·56	0·944	0·010	0·074	1·23	0·02–2·03	1·17	0·50–1·97	0·944
20:4*n*-6	8·53	7·13–10·35	8·53	7·73–9·33	1·000	16·07	13·88–17·62	15·20	14·42–17·59	0·832	0·000	0·005	7·20	5·73–9·25	7·36	6·65–8·93	0·796
20:5*n*-3	0·31	0·19–0·50	0·27	0·16–0·38	0·621	0·20	0·00–0·26	0·17	0·12–0·26	1·000	0·000	0·005	−0·16	−0·27 – −0·08	−0·09	−0·15 – −0·05	0·245
22:0	0·78	0·59–0·90	0·84	0·68–1·15	0·332	0·69	0·60–1·00	1·12	0·75–1·34	0·016	0·868	0·258	0·03	−0·24–0·25	0·25	−0·19–0·60	0·265
22:4*n*-6	0·29	0·16–0·33	0·28	0·25–0·42	0·226	0·63	0·46–0·94	0·54	0·50–0·62	0·408	0·000	0·007	0·37	0·23–0·62	0·28	0·18–0·31	0·109
22:5*n*-3	0·34	0·27–0·42	0·30	0·26–0·41	0·724	0·37	0·15–0·52	0·36	0·23–0·42	0·724	0·983	0·721	0·05	−0·17–0·10	0·01	−0·06–0·08	0·906
22:6*n*-3	4·32	3·17–5·07	2·29	2·12–3·11	0·005	6·44	3·60–7·53	3·97	2·94–4·93	0·003	0·001	0·005	2·03	0·27–3·55	1·16	0·40–2·47	0·524
24:0	0·43	0·26–0·56	0·52	0·47–0·60	0·097	0·56	0·40–0·76	0·81	0·71–0·88	0·040	0·030	0·022	0·23	−0·06–0·75	0·30	0·05–0·39	0·981
24:1*n*-9	1·62	1·17–1·86	1·22	1·03–1·29	0·121	1·11	0·81–1·74	1·23	1·09–1·84	0·332	0·088	0·386	−0·31	−0·63–0·44	0·12	−0·15–0·33	0·208
PL (mg/dl)	208·1	166·3–208·1	185·5	152·3–242·5	0·906	54·2	45·7–60·5	62·2	50·8–73·6	0·121	0·000	0·005	−53·88	−60·38 – −45·50	−62·05	−73·48 – −50·62	0·133

W., women; F., fetus; F.-M-, fetus–mother difference in mol%; FA, fatty acid.

DHA^+^: group with 200 mg/d of DHA supplementation.

DHA^–^: group without DHA supplementation.

* Inter group comparison made by Mann–Whitney *U* test.

† Intra group comparison made by Wilcoxon test.

At delivery, DHA content in maternal plasma phospholipids was significantly higher in the DHA^+^ group than in the DHA^–^ group, 4·32 (3·17–5·07) *v*. 2·29 (2·12–3·11) mol %, *P* = 0·005.

DHA content in cord plasma phospholipids was higher in the DHA^+^ group than in the DHA^–^ group (6·44 (3·60–7·53) *v*. 3·97 (2·94–4·93); *P* = 0·003). The content of C22:0 and C24:0 was significantly lower in cord plasma phospholipids of the DHA^+^ group compared to the DHA^–^ group.

Significant differences in FA mol% between mother and fetus can be viewed in Table 2. There were significant differences in the following long-chain PUFA: C18:2*n*-6, C18:3*n*-3, C20:3*n*-6, C20:4*n*-6, C20:5*n*-3, C22:4*n*-6 and C22:6*n*-3. We also found significant differences for C12:0, C18:0, C18:1*n*-9, C18:1*n*-7, C20:0 and C24:0.

**Fig. 1.**
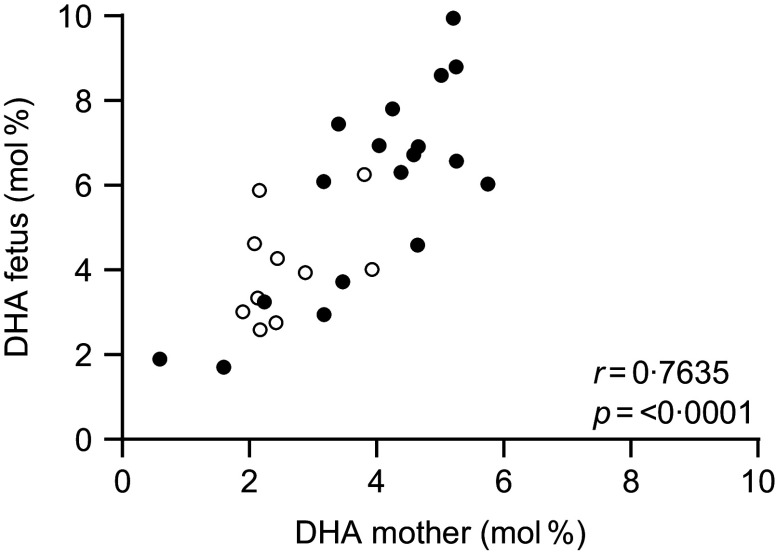
Correlation between maternal and fetal plasma phospholipids’ DHA in twenty-eight pregnant women receiving (filled dots) or not receiving (empty dots) DHA supplement.

We calculated the difference of the mol% values for each woman–fetal dyad for all the major FA and compared them to evaluate if the differences were influenced by DHA supplementation. We did not find any significant difference (Table 2). Figure 1 shows a significant correlation of DHA percentage in plasma phospholipids between mothers and their fetuses

### Maternal and cord blood plasma phospholipid fatty acids δ^13^C

**Fig. 2.**
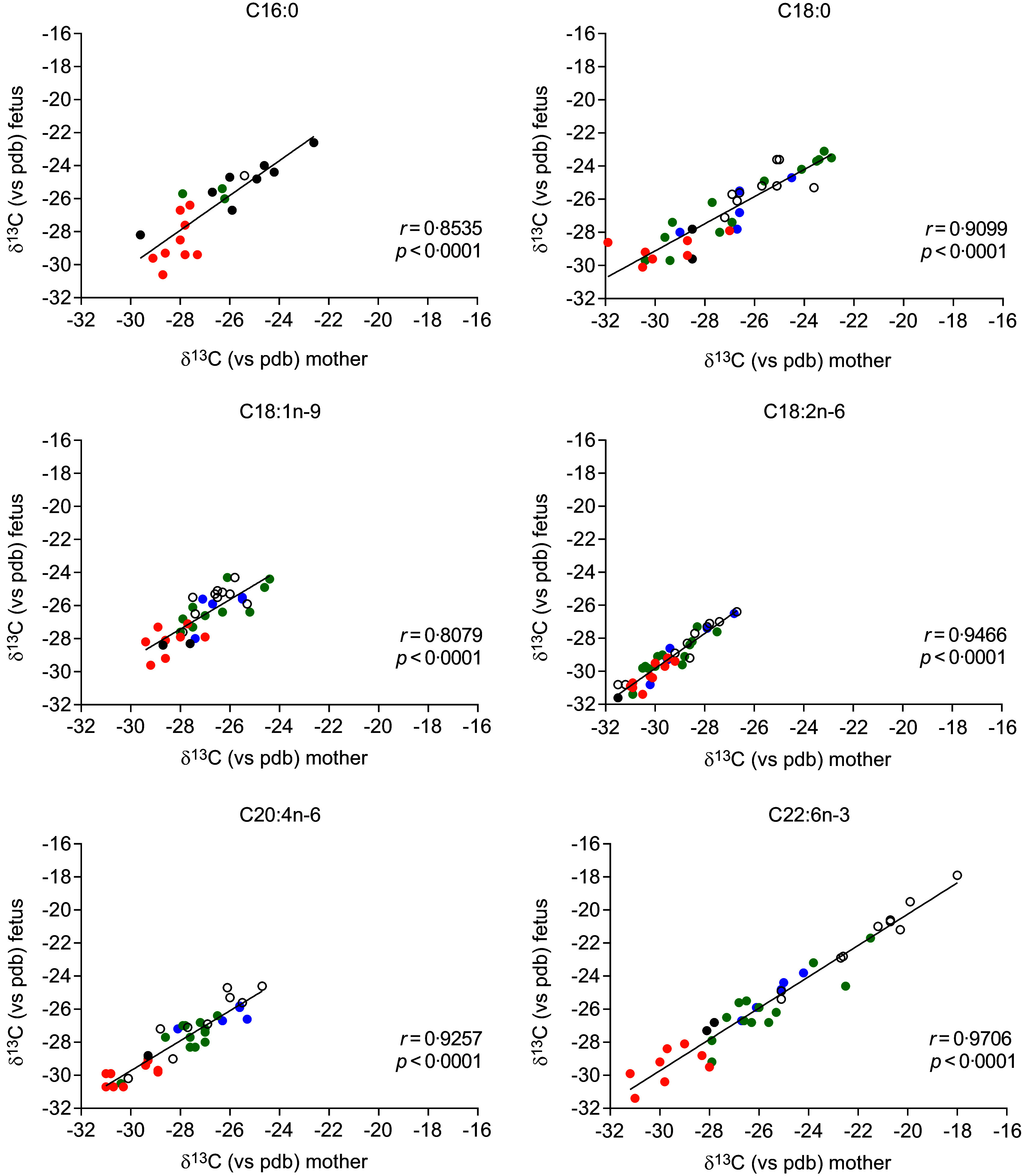
Correlation between the δ^13^C of the major plasma phospholipid FA measured in the mother and the fetus. The orange points are for the Dutch women, the empty points are for the DHA^–^ Italian Women, and the blue (fish-DHA) and the green (algal-DHA) points are for the DHA^+^ Italian women. Correlations were made by the Pearson test. FA, fatty acid.

The δ^13^C values of PA, OA, C18:1*n*-9; LA, AA and DHA isolated from maternal and cord plasma are illustrated in Fig. 2. Significant correlations were observed between the δ^13^C values of these FA in the plasma phospholipid fractions of mothers and their fetuses.

The δ^13^C value of plasma phospholipid DHA was significantly higher (*P* < 0·001) in women supplemented with DHA from algae (–21·9 (sd 2·1) mUr) compared with those who did not receive DHA supplementation (−27·6 (sd 2·1) mUr) or who were supplemented with DHA from fish oil (−27·1 (sd 0·9) mUr). Moreover, the Dutch women had the lowest ^13^C content (−29·0 (sd 1·3) mUr), possibly reflecting their diet.

**Fig. 3.**
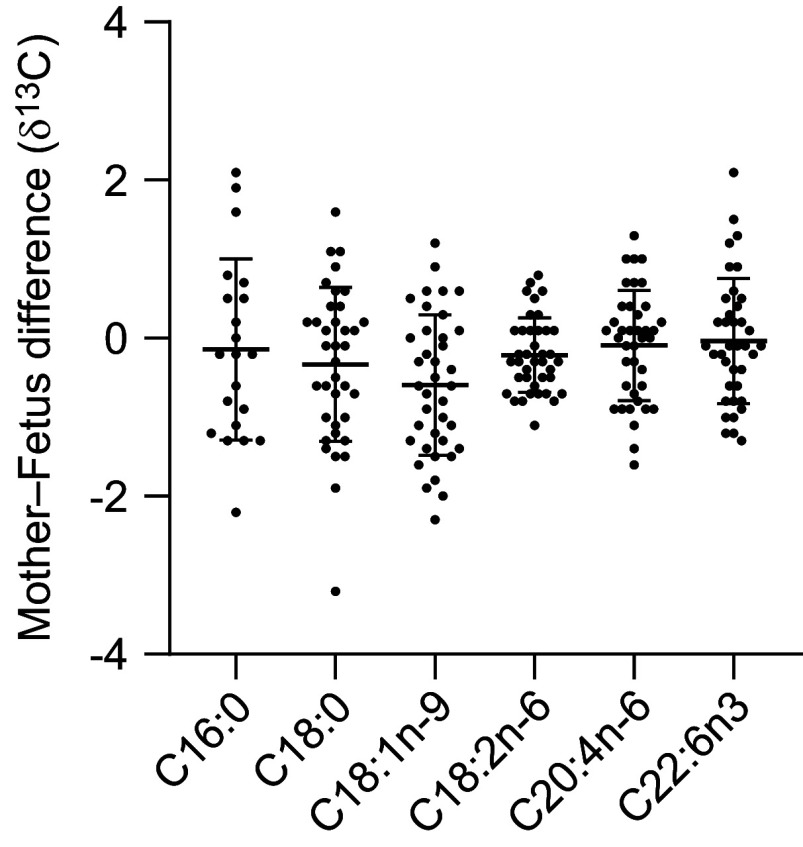
Differences between fetal and maternal δ^13^C of major FA. FA, fatty acid.

The differences in FA δ^13^C between mothers and fetuses are reported in Fig. 3.

The maternal–fetal difference of δ^13^C DHA is similar between DHA^+^ and DHA^–^ groups (*P* = 0·254).

## Discussion

In this study, we compared the δ^13^C values of selected FA between maternal and cord/fetal plasma phospholipids in uncomplicated pregnancies. We chose to analyse plasma phospholipids because they are rich in DHA. When designing this study, we hoped to demonstrate some differences between the δ^13^C values of selected FA of the woman and her fetus. Contrary to our expectations, we found: (1) a very close match between the δ^13^C values of all the major FA studied in the mother and their fetus and (2) no effect of the DHA supplementation on the difference in mol% values between maternal and fetal FA. These are important information that suggests a very efficient equilibration between the maternal and fetal FA, and a low or undetectable fetal biosynthesis of the major FA.

In line with other studies,^(27,28)^ we found that DHA^+^ women had higher DHA mol% values in their plasma phospholipids than DHA^–^ women and that the plasma DHA mol% values of the supplemented fetuses had significantly higher DHA mol% values than those belonging to DHA^–^ group. In line with this notion, Fig. 1 shows the correlation between maternal and fetal DHA mol% values.

The δ^13^C values of LA and AA were −29·4 (sd 1·3) mUr and −28·0 (sd 1·8) mUr in the mother and −29·2 (sd 1·4) mUr and −28·0 (sd 1·8) mUr in the fetuses. We can observe that the δ^13^C value of LA was nearly identical in the mother and her newborn. This result was expected and was reassuring as LA is an essential FA and no endogenous biosynthesis is expected neither in the mother nor in her fetus. The δ^13^C value found in plasma phospholipids likely reflects the ^13^C of LA content of the maternal diet (not measured in this pilot study) and likely indicates a condition of steady state, as we asked the pregnant women to maintain their diet constant in terms of the sources of fats and oils. In our study, the mean difference in δ^13^C between LA from pregnant women (DHA^–^ group) and their fetuses was −0·2 mUr with maximum and minimum values of 0·8 and −0·8 mUr, respectively. From these data, we extrapolated the error of our method (analytical plus biological variance) to be 0·8 mUr based on the maximum difference between maternal and fetal plasma δ^13^C LA.

The mean difference of δ^13^C values of AA between pregnant women and their fetuses was −0·1 mUr with a range from a maximum of 1·3 mUr to a minimum of −1·6 mUr. The mean differences between δ^13^C values of phospholipid LA (precursor) and AA (product) were 1·2 mUr (max 4·2, min −1·1 mUr) in fetuses and 1·3 mUr (maximum 4·9, minimum −1·3 mUr) in mothers. The difference between these values was close to zero. If there were significant endogenous biosynthesis of AA from LA, we would have expected some, consistent δ^13^C difference between the precursor and product, which was not observed. Only ten out of thirty-nine mother–fetus pairs had an AA δ^13^C difference greater than the maximum error of our method. These findings suggest that the AA supply to the fetus was far larger than any endogenous biosynthesis if it occurs at all^(29)^. We cannot exclude, however, that the acetyl molecules incorporated during chain elongation had the same δ^13^C of LA. In both fetuses and neonates, the main precursor of lipogenesis is glucose^(30)^. In Europe, carbohydrates like potatoes and bread/wheat are the main sources of glucose. Both have C3 signature, with low ^13^C content (–24 < δ^13^C < –30 mUr). Unfortunately, we did not measure plasma glucose δ^13^C nor δ^13^C of the carbohydrates of women’s diet. This made it difficult to calculate the contribution, in terms of ^13^C of the acetyl molecules, derived from glucose, incorporated during chain elongation. It is also possible that our method was not sensitive enough to detect a trivial endogenous biosynthesis of AA as the variation in enrichment was determined by the addition of two carbon atoms out of twenty, and only in the case that the added carbon atoms were different in C^13^ from those of the parent FA, namely LA. In this scenario, it would be impossible to calculate AA fetal biosynthesis in the absence of an AA supplement with a different δ^13^C than that of LA. Such a study would be feasible with the use of an AA supplement with a high content of ^13^C. Obtaining information on AA is important, as AA is the second most represented long-chain PUFA in the brain,^(31)^ and data on its perinatal metabolism are scanty, especially in humans.

The mean difference of δ^13^C values of DHA between pregnant women and their fetuses was +0·2 mUr in the DHA^+^ group and −0·2 mUr in the DHA^–^ group. Notably, in the women receiving algal DHA (usually with higher ^13^C content), the maternal–fetal DHA δ^13^C difference was below our methodological error for all study dyads, but one pair (–2·9 mUr). These findings strongly suggest a marked dependency of the human fetus from the maternal long-chain PUFA supply, with no significant fetal biosynthesis as reported in animal studies^(29)^.

Stable isotope studies in preterm infants have demonstrated that premature infants can synthesise both AA and DHA,^(21,32,33)^ but the synthesis is not sufficient to meet their high nutritional requirements^(34)^. Information on endogenous biosynthesis in the human fetus, especially in the presence of a likely ‘abundant’ maternal supply, is not available so far.

We were surprised to find very low δ^13^C differences for PA and OA between mothers and their fetuses. Given that fetal lipogenesis is known to occur, with some lipid synthesis detected in adipose tissue from 10 weeks onwards^(16)^, we anticipated more pronounced differences.

The mean δ^13^C difference for PA between pregnant women and their fetuses at delivery was −0·1 mUr with a maximum of 2·1 mUr and a minimum of −2·2 mUr. For OA, the mean difference was −0·3 mUr with a maximum of 1·6 mU and a minimum of −3·2 mUr. We expected some consistent differences between the mean of the maternal–fetal differences of PA *v*. OA or at least some random differences in individual patients. We found no statistically significant differences between the maternal–fetal differences of PA and OA compared with LA (*P* = 0·818 and *P* = 0·544, respectively, by Wilcoxon test).

Our pilot study could not demonstrate the endogenous fetal biosynthesis of PA and OA so, we conclude that in our subjects this was very low or absent. An alternative hypothesis could be that lipogenesis does occur at the cellular level (mainly in adipose tissue which most studies focused on) with limited exchange with plasma lipids.

This point requires a few additional considerations. If, from one side, it could be hypothesised that the same glucose was used for lipogenesis in the mother and the fetus (well-known dependency of fetal glucose from maternal supply),^(35)^ it is also true that lipogenesis in the last trimester of pregnancy is low^(4)^ and most likely the ^13^C enrichment of plasma phospholipid PA in maternal plasma is from dietary origin. We thought that if fetal PA biosynthesis from glucose was to be more active than in pregnant women, this would have resulted in a more evident maternal–fetal difference.

In addition, in agreement with previous studies,^(36,37)^ our findings indicate that a daily supplementation of 200 mg of DHA in the second half of pregnancy significantly increases the DHA percentage in maternal plasma phospholipids. Our stable isotope study demonstrated that maternal DHA supplementation (richer in ^13^C) increased the DHA enrichment of the fetus indicating the transfer and incorporation of DHA from dietary supplement into the fetal plasma phospholipids.

Despite the limited sample size our data shows that 200 mg/d of DHA from algae did not reduce AA levels in both mothers and fetuses.

In conclusion, this pilot study did not show clear evidence of fetal synthesis of major FA including saturated, unsaturated, the essential LA and long-chain PUFA AA and DHA.

Our study has some limitations. First, we did not measure plasma palmitate and glucose levels, the main sources of acetyl CoA for maternal and fetal lipogenesis. Second, we asked the pregnant women to maintain a constant diet, but we did not ask them to compile a food diary. Third, the limited number of patients makes it impossible to look at differences linked to the type of delivery and fetus gender.

Future studies using our non-invasive method with a larger number of patients may confirm these preliminary findings or provide a deeper understanding of the fetal dependency on placenta FA transport in healthy pregnant women on a normal diet, as well as in cases of nutrition disturbances or maternal diseases.
