# Fetal dependency on maternal fatty acids: a pilot study in human pregnancies using the natural abundance variation of 13C – CORRIGENDUM

**DOI:** 10.1017/S0007114525105576

**Published:** 2026-01-14

**Authors:** Manuela Simonato, Giovanna Verlato, Silvia Visentin, Erich Cosmi, Anna Sartori, Pieter Sauer, Alessio Correani, Paola Cogo, Virgilio Carnielli

In the original publication multiple author names were formatted incorrectly. The correct author list is as follows:

Manuela Simonato, Giovanna Verlato, Silvia Visentin, Erich Cosmi, Anna Sartori, Pieter Sauer, Alessio Correani, Paola Cogo and Virgilio Carnielli.

This has been updated in the original article.
